# Antibody response to BNT162b2 SARS-CoV-2 mRNA vaccine in adult patients with systemic sclerosis

**DOI:** 10.1007/s10067-022-06219-7

**Published:** 2022-05-26

**Authors:** Chiara Pellicano, Roberta Campagna, Alessandra Oliva, Giorgia Leodori, Marzia Miglionico, Amalia Colalillo, Ivano Mezzaroma, Claudio Maria Mastroianni, Ombretta Turriziani, Edoardo Rosato

**Affiliations:** 1 Department of Translational and Precision Medicine, Sapienza University of Rome, Viale dell’Università 37, 00185 Rome, Italy; 2 Department of Molecular Medicine, Sapienza University of Rome, Rome, Italy; 3 Department of Public Health and Infectious Diseases, Sapienza University of Rome, Rome, Italy

**Keywords:** COVID-19, Humoral response, SARS-CoV-2, Systemic sclerosis, Vaccination

## Abstract

**Objectives:**

Systemic sclerosis (SSc) patients are at risk for a severe disease course during SARS-CoV-2 infection either due to comorbidities or immunosuppression. The availability of SARS-CoV-2 vaccines is crucial for the prevention of this hard-to-treat illness. The aim of this study is to assess the humoral response after mRNA vaccination against SARS-CoV-2 in SSc patients.

**Method:**

Seropositivity rate and serum IgG levels were evaluated 1 month (t1) and 3 months (t3) after the second dose of vaccine in a cohort of SSc patients and healthy controls (HC). Differences were made with Student’s or Mann–Whitney’s *t*-test and with the chi-square or Fisher exact test. Logistic regression model including immunosuppressive treatments (corticosteroids, CCS; mycophenolate mofetil, MMF; methotrexate, MTX; rituximab, RTX) was built to assess the predictivity for seropositivity.

**Results:**

The seropositivity rate was similar in 78 SSc patients compared to 35 HC at t1 but lower at t3. SSc patients had lower serum IgG levels than HC at t1 but not at t3. SSc patients treated with immunosuppressive therapy showed both a lower seropositive rate (t1, 90.3% vs 100%; t3, 87.1% vs 97.9%; *p* < 0.05) and serum IgG levels than untreated patients both at t1 [851 BAU/ml (IQR 294–1950) vs 1930 BAU/ml (IQR 1420–3020); *p* < 0.001] and t3 [266 BAU/ml (IQR 91.7–597) vs 706 BAU/ml (IQR 455–1330); *p* < 0.001]. In logistic regression analysis, only MTX was significant [OR 39.912 (95% CI 1.772–898.728); *p* < 0.05].

**Conclusions:**

SSc patients treated with MTX had a lower serological response to mRNA vaccine, and even low doses of CCS can adversely affect antibody titer and vaccination response.

**Table:** 

**Key Points** *• SSc patients are able to produce vaccine-induced antibodies after mRNA vaccination.* *• In SSc patients, clinical characteristics of disease did not influence seropositivity rate.* *• In SSc patients, even low doses of CCS can adversely affect antibody titer and vaccination response.* *• In SSc patients, MTX treatment is mainly associated with reduced seropositivity and lower serum IgG levels.*

## Introduction

Severe acute respiratory syndrome coronavirus-2 (SARS-CoV-2) was first reported in late December 2019, was recognized as the causative agent of an acute respiratory tract infection named Coronavirus Disease 2019 (COVID-19) and was then declared as a pandemic on 10th March 2020 [1].

The majority of patients are asymptomatic or exhibit mild respiratory symptoms; nevertheless, hospitalization and admission to Intensive Care Unit (ICU) may occur in up to 10% of cases [2]. Apart from age, sex and comorbidities [3, 4], the immunosuppressive drugs, pose patients at higher risk of severe form of infection and mortality from COVID-19 [5–7].

In this scenario, the availability of SARS-CoV-2 vaccines represented a crucial step for reducing severe infections, hospitalization and mortality [8], and, given their vulnerability to SARS-CoV-2 severe form of infection, international guidelines called for priority vaccination of immunosuppressed patients.

Patients with immunosuppression experience lower and less durable response to vaccination than immunocompetent subjects, and therefore additional vaccine injections as well as individual immune monitoring may be required [9–14].

Several studies investigated the safety of messenger RNA (mRNA) vaccines in patients with autoimmune inflammatory diseases and the effects of immunosuppressive therapy on vaccine efficacy in this cohort of patients [15–23]. No data are reported about the only cohort of systemic sclerosis (SSc) patients vaccinated with mRNA vaccines. To date, only one study focuses exclusively on SSc patients undergoing inactivated SARS-CoV-2 vaccine [24]. SSc is an autoimmune disease characterized by dysregulation of immune system, vascular damage and fibrosis of skin and internal organs leading to challenging comorbidities [25]. Moreover, immunosuppressive therapies (i.e., corticosteroids, CCS; mycophenolate mofetil, MMF; methotrexate, MTX; rituximab, RTX) are used to slow down or stabilize the major and fearful complications of SSc, such as interstitial lung disease (ILD) [26]. The outbreak of COVID-19 has radically changed the quality of life of SSc patients [27]. SSc patients may be at risk for a severe disease course either due to underlying ILD and/or immunosuppression when they develop SARS-CoV-2 virus infection [28]. The availability of SARS-CoV-2 vaccines represents one of the safest and most effective means to prevent this hard-to-treat illness. In a recent large cross-sectional multicentric study, conducted by the international Scleroderma Patient-centered Intervention Network (SPIN) Cohort, the vaccination was safe in this group with no serious adverse events, a side-effect profile similar to that seen in other populations, and a low rate of reported SSc flare [29].

Aim of this study is to assess the humoral response after two doses of mRNA vaccine against SARS-CoV-2 in a cohort of SSc patients.

## Materials and methods

### Subjects

Ninety SSc patients, fulfilling the American College of Rheumatology/European League Against Rheumatism Collaborative Criteria for SSc [30], and 58 HC, matched for sex and age, were enrolled in this study. All study participants were administered the two dose regimen BNT162b2 mRNA vaccine (Pfizer-BioNTech), 30 mcg per dose, by intramuscular injection 3 weeks apart, as indicated by the national guidelines. SSc patients continued all medications, except for MTX which was stopped one week before and one week after vaccine administration, following the recommendations of the major scientific society of rheumatology [28, 31].

Exclusion criteria were prior SARS-CoV-2 infection, patients receiving only one dose of vaccine, age < 18 years, and immunosuppressive therapy with RTX in the last 6 months before vaccination.

The subjects’ written consent was obtained, and the study was conducted according to the Declaration of Helsinki. The study was approved by the ethics committee of Sapienza University (IRB n 0486).

### Laboratory assays

Peripheral venous blood was collected at two different time points: 1 (t1) and 3 (t3) months after the second vaccine dose. Serum was separated from blood cells by centrifugation at 1500 × *g* for 15 min at room temperature and stored at − 70 °C until test was performed. The two samples collected at the two different time points from each individual were concomitantly thawed and tested with LIAISON® SARS-CoV-2 TrimericS IgG assay to measure anti SARS-CoV-2 IgG.

### Clinical assessment of SSc patients

Modified Rodnan skin score (mRSS) and disease subset (limited cutaneous SSC, lcSSc, or diffuse cutaneous SSc, dcSSc) were evaluated [32]. Digital ulcers (DUs) were defined as Amanzi et al. [33]. Disease duration (time from first non-Raynaud manifestation), disease activity index (DAI), and disease severity scale (DSS) were assessed following EUSTAR indications [34, 35]. NVC was performed at the level of the distal phalanx of the second, third, and fourth fingers of both hands using a videocapillaroscope equipped with a 500 × magnification lens (Pinnacle Studio Version 8 software), and the capillaroscopic images have been classified in the patterns: early, active, and late, according to Cutolo et al. [36]. SSc patients were evaluated to estimate pulmonary arterial hypertension (PAH), according to ESC/ERS guidelines, by echocardiography and/or right hearth catheterization (RHC) and ILD by pulmonary function tests (PFTs) and/or high resolution computed tomography (HRCT) according to the standards recommended by the American/European Respiratory Society [37, 38].

### Statistical Analysis

SPSS version 26.0 software was used for statistical analysis. After evaluation of normality, continuous variables were expressed as median and interquartile range (IQR). Student’s or Mann–Whitney’s *t*-test was used to evaluate differences between groups. Bonferroni’s corrections were applied in case of multiple comparisons. The chi-square or Fisher exact test was used to evaluate differences between categorical variables. The Pearson or Spearman correlation test was used for bivariate correlations. Logistic regression model was built to assess the predictivity of continuous or categorical variable (CCS, MMF, MTX, RTX) for a dichotomic dependent variable, expressed as odds ratio (OR) and 95% confidence interval (95% CI). A *p* value < 0.05 was considered significant.

## Results

Statistical analysis was performed in 78 SSc patients [F = 65 (83.3%), median age 50 years (IQR 36–61 years)] and 35 HC, due to missing serology tests. Demographic and clinical features of SSc patients are shown in Table 1.

**Table 1:** Demographic and clinical features of 78 SSc patients

Age, years, median and IQR	50 (36–61)
Female, *n* (%)	65 (83.3)
dcSSc, *n* (%)	33 (42.3)
Disease duration, years, median and IQR	13 (7–16)
mRSS, median and IQR	11 (8–16)
SSc-specific autoantibodies	
Anti-topoisomerase I, *n* (%)	27 (34.6)
Anti-centromere, *n* (%)	21 (26.9)
Anti-RNApolymerase III, *n* (%)	2 (2.6)
None, *n* (%)	28 (35.9)
Nailfold capillaroscopic pattern	
Early, *n* (%)	16 (20.5)
Active, *n* (%)	20 (25.6)
Late, *n* (%)	42 (53.8)
DAI, median and IQR	1.42 (0.76–2.5)
DSS, median and IQR	4 (2–5)
DUs’ history, *n* (%)	42 (53.8)
Active DUs, *n* (%)	6 (7.7)
ILD, *n* (%)	61 (78.2)
PAH, *n* (%)	7 (9)
Immunosuppressive therapies, *n* (%)	31 (39.7)
Prednisone or equivalent	
5 mg/die, *n* (%)	16 (20.5)
10 mg/die, *n* (%)	13 (16.7)
MTX, *n* (%)	6 (7.7)
MMF, *n* (%)	4 (5.1)
RTX*, *n* (%)	8 (10.3)

*SSc* systemic sclerosis, *dcSSc* diffuse cutaneous systemic sclerosis, *mRSS* modified Rodnan skin score, *DAI* disease activity index, *DSS* disease severity scale, *DUs* digital ulcers, *ILD* interstitial lung disease, *PAH* pulmonary arterial hypertension, *MTX* methotrexate, *MMF* mycophenolate mofetil, *RTX* rituximab, *IQR* interquartile range. *SSc patients treated with RTX > 6 months before first dose of vaccine

### Seropositivity rate and serum IgG levels in SSc patients and HC

**Fig. 1:**
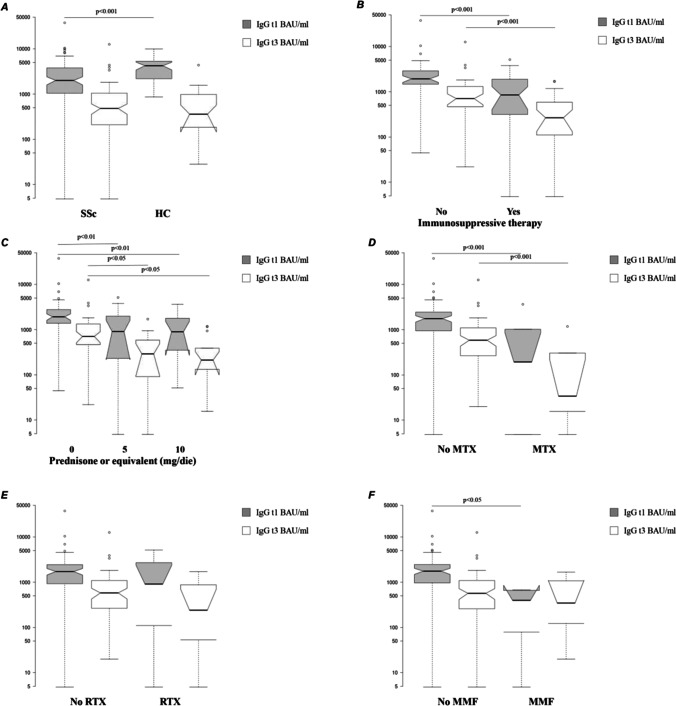
Anti-spike IgG levels 1 month (t1) and 3 months (t3) after the second dose of vaccine. Anti-spike IgG levels in SSc and HC (**A**). Anti-spike IgG levels in treated and untreated SSc patients (**B–F**). SSc, systemic sclerosis; HC, healthy controls; MTX, methotrexate; RTX, rituximab; MMF, mycophenolate mofetil

Table 2 summarizes Ab positivity rate and serum IgG levels in SSc patients and HC. Figure 1A shows the median serum IgG levels at t1 and t3 in SSc patients and HC.

**Table 2:** Immunogenicity expressed as seroconversion rate and anti-spike IgG levels of the mRNA BNT162b2 vaccine in SSc patients and HC

	HC (*n* = 35)	SSc (*n* = 78)	*p*
Ab t1 positivity rate, *n* (%)	35 (100)	75 (96.2)	> 0.05
Ab t3 positivity rate, *n* (%)	35 (100)	73 (93.6)	< 0.05
IgG t1, BAU/ml, median, and IQR	4238 (2119–5382)	1705 (851–2440)	< 0.001
IgG t3, BAU/ml, median, and IQR	358.8 (177.06–1021.8)	557.5 (225–1090)	> 0.05

*SSc* systemic sclerosis, *HC* healthy controls, *Ab* antibodies, *IQR* interquartile range

In a sub-analysis, comparing only SSc patients without immunosuppressive therapy (IT) (*n* = 47) with HC, the seropositivity rate was similar at both t1 and t3 (100% vs 100% and 99% vs 100%; *p* > 0.05). SSc patients without IT had a statistically significant lower serum IgG levels than HC at t1 [1930 BAU/ml (IQR 1420–3020) vs 4238 BAU/ml (IQR 2119–5382); *p* < 0.001], but at t3, the serum IgG levels was significantly higher in SSc patients without IT than HC [706 BAU/ml (IQR 455–1330) vs 358.8 BAU/ml (IQR 177.06– 1021.8); *p* < 0.01].

During the study period, a total of 11 (14.1%) patients developed SARS-CoV-2 infection, after a median of 7 months (IQR 7–8) from the first dose of vaccine. Six of these 11 patients (54.5%) were in treatment with IT, and only one of them died consequently to SARS-CoV-2 infection. SSc patients who developed SARS-CoV-2 infection had significantly lower serum IgG levels at t1 [928 BAU/ml (IQR 385–1390) vs 1820 BAU/ml (IQR 966–2770); *p* < 0.01] but not at t3 [335 BAU/ml (IQR 90.4–674) vs 597 BAU/ml (IQR 266–1220); *p* > 0.05] compared to SSc patients who did not developed SARS-CoV-2 infection.

Seropositivity rate was similar according all clinical characteristics analyzed both at t1 and t3 (*p* > 0.05). There was no correlation between antibodies titer and age, disease duration, and any disease feature (i.e., mRSS, DAI, DSS), and serum IgG levels were similar according gender and clinical characteristics of disease (i.e., disease subset, NVC pattern, autoantibody specificity, presence or history of DUs, PAH, ILD). No patients experienced flares of disease after vaccination.

### Seropositivity rate and serum IgG levels in SSc patients according to IT

Overall, SSc patients treated with IT showed both a lower seropositive rate (t1, 90.3% vs 100%; t3, 87.1% vs 97.9%; *p* < 0.05) and serum IgG levels than untreated patients both at t1 [851 BAU/ml (IQR 294–1950) vs 1930 BAU/ml (IQR 1420– 3020); *p* < 0.001] and t3 [266 BAU/ml (IQR 91.7–597) vs 706 BAU/ml (IQR 455–1330); *p* < 0.001]. Figure 1B shows the median serum IgG levels in SSc patients in treatment with IT and SSc untreated patients.

In order to evaluate the effect of the different therapeutic regimens on vaccine response, patients were stratified according to treatment received.

In SSc patients in treatment with steroid, the seropositivity rate at t1 and t3 was significantly lower compared to SSc patients not in steroid treatment (t1, 34.7% vs 65.3%; t3, 34.2% vs 65.8%; *p* < 0.05). Moreover, serum IgG levels were significantly lower in SSc patients in treatment with 10 mg/daily or 5 mg/daily of prednisone or equivalent compared to SSc patients not in treatment with steroid both at t1 [897 BAU/ml (IQR 350–1780) vs 1920 BAU/ml (IQR 1380– 2770) or 908.5 BAU/ml (IQR 231.5–1985) vs 1920 BAU/ml (IQR 1380– 2770), respectively; *p* < 0.01] and t3 [212 BAU/ml (IQR 132–389) vs 706 BAU/ml (IQR 467– 1330) or 290.5 BAU/ml (IQR 91.05–585) vs 706 BAU/ml (IQR 467– 1330), respectively; *p* < 0.05]. No significantly difference in serum IgG levels were detected between SSc patients in treatment with 10 mg/daily of prednisone or equivalent compared to SSc patients in therapy with 5 mg/daily of prednisone or equivalent both at t1 [897 BAU/ml (IQR 350–1780) vs 908.5 BAU/ml (IQR 231.5–1985); *p* > 0.05] and t3 [212 BAU/ml (IQR 132–389) vs 290.5 BAU/ml (IQR 91.05– 585); *p* > 0.05]. Figure 1C shows the median serum IgG levels in these groups of patients.

In SSc patients treated with MTX, the seropositivity rate at t1 and t3 was significantly lower compared to untreated patients (t1 66.7% vs 98.6%; t3, 50% vs 97.2%; *p* < 0.001). Serum IgG levels at t1 [193.55 BAU/ml (IQR 4.81–1020) vs 1755 BAU/ml (IQR 947–2450); *p* < 0.001] and t3 [33.95 BAU/ml (IQR 15.6–304) vs 585 BAU/ml (IQR 266.5–1095); *p* < 0.001] were significantly lower in MTX treated patients than in untreated patients (Fig. 1D).

In our study population, the mean time since the last rituximab infusion was 12 ± 1.5 months. Seropositivity rate was similar between SSc patients in treatment with RTX compared to SSc patients not in treatment with RTX both at t1 (87.5% vs 97.1%; *p* > 0.05) and t3 (75% vs 95.7%; *p* > 0.05). Moreover, serum IgG levels were similar between SSc patients in treatment with RTX compared to SSc patients not in therapy with RTX both at t1 [909.5 BAU/ml (IQR 110.05–2710) vs 1720 BAU/ml (IQR 928–2440); *p* > 0.05] and t3 [240.35 BAU/ml (IQR 53–876.5) vs 579.5 BAU/ml (IQR 266–1090); *p* > 0.05]. Figure 1E shows the median serum IgG levels in SSc patients treated with RTX and SSc untreated patients.

In SSc patients under MMF treatment, the seropositivity rate at t1 was significantly lower compared to SSc patients not in treatment with MMF (75% vs 97.3%; *p* < 0.05). Moreover, SSc patients in therapy with MMF serum IgG levels at t1 were significantly lower than SSc patients not in treatment with MMF [397 BAU/ml (IQR 78.91–658.5) vs 1755 BAU/ml (IQR 966–2460); *p* < 0.05]. Conversely, similar seropositivity rate at t3 were observed between MMF treated and untreated patients (75% vs 94.6%; *p* > 0.05), and also serum IgG levels were similar between these groups [346 BAU/ml (IQR 122.45–1073.5) vs 567.5 BAU/ml (IQR 259–1090); *p* > 0.05] (Fig. 1F).

In logistic regression analysis all immunosuppresive drugs were included, but only MTX was significantly associated to non-response to vaccination [OR 39.912 (95% CI 1.772—898.728); p < 0.05). Table 3 summarizes the regression model analysis.

**Table 3:** Logistic regression analysis to assess the predictivity of treatment for a non-response to vaccination, expressed as odds ratio (OR) and 95% confidence interval (95% CI)

	OR (95% CI)	*p*
Corticosteroids	1.362 (0.063–29.322)	> 0.05
Methotrexate	39.912 (1.772–898.728)	< 0.05
Rituximab	2.347 (0.139–39.629)	> 0.05
Mycophenolate mofetil	23.644 (1.088–513.767)	> 0.05

## Discussion

This study focuses on efficacy and immunogenicity of BNT162b2 mRNA SARS-CoV-2 vaccine in SSc patients. In this cohort of patients, the seropositivity conversion was similar 1 month after the second injection but significantly lower 3 months after the completion of the vaccination cycle compared to HC. Newsworthy, the antibody titer was lower at t1 and similar at t3 between SSc patients and HC. Hence, SSc patients were able to produce vaccine-induced antibodies after mRNA vaccination and clinical characteristics of disease did not influence seropositivity rate in this cohort of patients, according to previous study [24]. In the same way, vaccination does not seem to have influenced, in this short observation period, the course of the disease as no patient has experienced flares of disease. Other studies evaluated the drop at t3 in healthy controls. Brisotto et al. in 516 healthy healthcare workers at t3 demonstrated a serological response decay from 559.8 AU/ml to 92.7 AU/ml with a titer reduction of approximately 6 times [39]. In our study, using a different kit, we demonstrated a titer reduction in healthy healthcare workers of 11 times. Although the titer reduction in our population is wider, even larger studies using other analytical methods found a significant titer reduction at t3.

In this study, a total of 11 (14.1%) patients developed SARS-CoV-2 infection, after a median of 7 months from the first dose of vaccine; nevertheless, only one patient had a severe form of infection, and this confirms the beneficial effect of vaccine. Moreover, SSc patients who developed SARS-CoV-2 infection after vaccination had significantly lower antibodies titre at t1 compared to SSc patients who did not develop SARS-CoV-2 infection, suggesting a protective role of the antibody response, although not exclusive.

Overall, SSc patients treated with immunosuppressive therapy showed both a lower seropositive rate and serum IgG levels than untreated patients. This is in line with the literature showing that patients with immunosuppression (either for the underlying disease or for immunosuppressive therapy) exhibit a lower response to vaccination than immunocompetent subjects, despite also that non-immunocompromised individuals showed a decreased rate of seropositivity and antibodies levels after 3 months from the second dose of vaccination [40].

As a general concept, SARS-CoV-2 vaccines are able to induce specific cellular and humoral responses generating immune memory and therefore preventing both infection and its severe form. Nevertheless, when these strictly related immunological processes (i.e., innate immune activation signals, interactions between CD4 + T cells and activated B cells, production of antibodies) are impaired as a consequence of disease or immunosuppressive therapy, the development of protective immunity may be absent and, if present, delayed [41].

So far, immunocompromised individuals have been hardly included in the registrative studies investigating the efficacy of SARS-CoV-2 mRNA vaccines, and therefore the currently available data mostly come from subsequent real world studies [42].

As a matter of fact, Bergmann et al. performed a prospective clinical trial investigating the humoral response after 2 weeks from the second dose of mRNA vaccination in different immunocompromised patients, including primary or secondary immunodeficiency disorders (human immunodeficiency virus infection, allogeneic hematopoietic stem cell transplantation/CAR T cell therapy, solid organ transplantation, chronic lymphocytic leukemia) and found that the rate of seroconversion was further lower in these groups of patients compared to HC, with the lowest response in individuals suffering from solid organ transplantation and chronic lymphocytic leukemia. Of note, authors found that receiving immunosuppressive therapy with MMF and ibrutinib was independently associated with absence of seroconversion [43].

Likewise, subjects undergone cardiothoracic transplant had impaired humoral and cellular response early after the second dose of vaccine [44], whereas in a small group of lung transplant subjects, the seroconversion rate was even absent [45].

A recent meta-analysis showed that the proportion of vaccine non-responders was higher in solid organ transplant recipients and patients with hematological malignancies and lower in patients with cancer and those on dialysis, with risk factors for non-response including older age, corticosteroids, other immunosuppressive therapies such as MMF and MTX, or anti-CD20 agents [46].

On the other hand, the beneficial effect of additional doses of vaccination in patients with solid tumors has been demonstrated by Shroff et al., who showed that neutralizing antibodies progressively raised after the second and the third doses [47].

Although SSc patients are treated with prednisone doses of less than 10 mg/daily, this study found that even low doses of CCS can adversely affect antibody titer and vaccination response.

In this study, MTX treatment was mainly associated with reduced seropositivity and lower serum IgG levels compared to HC, suggesting that the withdrawal period is too short not to influence the response to the vaccine, probably due to reduced T-cell-mediated vaccine-induced immunity [23]. In contrast with other data in literature, in this study, RTX treatment did not influence the antibodies response to vaccination, probably due to the degree of B cell recovery at the time of vaccination [15, 22].

According to the data in the literature, treatment with MMF was associated with a reduced response to vaccination and a lower antibody titer [16].

Main limitations of this study included the monocentric design, small sample size in treatment groups, the randomization 2:1 for HC, the lack of serological data between the first and the second dose of vaccine, the lack of lymphocyte typing to better characterize the immune response, and the short serological follow-up.

In conclusion, this study showed how BNT162b2 mRNA SARS-CoV-2 vaccine is safe in SSc patients and how effective it is in preventing severe forms of the COVID-19 disease. Moreover, this study showed how SSc patients treated with MTX have a minor antibody response, suggesting a personalized management of preventive measures.

## Data Availability

The datasets generated during and/or analyzed during the current study are available from the corresponding author on reasonable request.
